# Estimating behavioral multipliers to American black duck and lesser scaup resting metabolic rate to better estimate daily energy expenditure

**DOI:** 10.1007/s00360-026-01672-1

**Published:** 2026-06-22

**Authors:** Jacob W. McPherson, Christopher K. Williams, Alicia M. Berlin, John M. Coluccy

**Affiliations:** 1 Department of Entomology and Wildlife Ecology, University of Delaware, 531 South College Ave, Room 250 Townsend Hall, Newark, DE 19716 USA; 2 U.S. Geological Survey, Patuxent Wildlife Research Center, 12100 Beech Forest Road, Laurel, MD 20708 USA; 3 Ducks Unlimited Incorporated, 7322 Newman Boulevard, Building 1, Dexter, MI 48130 USA

**Keywords:** Behavior, Energetic carrying capacity, Resting metabolic rate, Waterfowl

## Abstract

Historic declines of American black duck (*Anas rubripes*, hereafter black duck) and lesser scaup (*Aythya affinis*) populations may be the result of multiple factors including resource availability on migration and wintering landscapes. Bioenergetics modeling is used to calculate the carrying capacity of a landscape by estimating energy demand and energy supply. Past estimates of resting metabolic rate (RMR) and behavioral specific multipliers to RMR, physiologic parameters required for calculating energetic demand, are limited and, in some cases, based upon dated past research. We used open-flow respirometry techniques to estimate RMR and behavioral specific multipliers to RMR for captive black ducks (*n* = 6) and lesser scaup (*n* = 6) at U.S. Geological Survey’s Patuxent Wildlife Research Center in Laurel, Maryland during winter 2015/2016. RMR estimates for black ducks (357.28 kJ/bird/day) and lesser scaup (381.43 kJ/bird/day) in this study fell within the range of previously published estimates for each species. Behavioral multiplier estimates were different between species and from those reported in previous studies. We found that similarities between multiplier estimates for certain behaviors in each species justified combining behaviors into homogenous groups representing high, medium, low, and extreme low (or RMR) energy behavior classes. We tested the effects of six covariates (time of day [AM/PM], presence of live or decoy cohort, food presence [Y/N], weight [g], air temperature [C], and water temperature [C]) on energy expenditure within each behavioral grouping and within each species. Black ducks engaged in “medium” and “high-energy behaviors” experienced increasing energetic demand as water temperature decreased, and energetic demand interactively increased with higher energy behaviors. Energy expenditure increased with increasing mass in both black ducks and lesser scaup engaged in “high-energy behaviors.” Lesser scaup engaged in “medium-energy behaviors” expended more energy in the morning than in the afternoon. Lesser scaup engaged in “high-energy behaviors” expended more energy when food was absent than when present. Results from this study technologically improve past estimates, refine estimates derived using allometric equations, simplify the underlying calculations required for bioenergetics modeling and can help improve the accuracy of landscape carrying capacity estimates resulting from those models.

**Supplementary Information:**

The online version contains supplementary material available at 10.1007/s00360-026-01672-1.

## Introduction

Waterfowl comprise one of the most observed and economically important groups of wildlife in North America (U.S. Department of the Interior et al. 2011, North American Waterfowl Management Plan Committee [NAWMP] 2012). Accordingly, to ensure a sustainable waterfowl resource, considerable effort has been invested toward understanding waterfowl ecology at a continental scale (NAWMP 1986). The majority of waterfowl are showing stable or increasing populations (NAWMP 2012), but the American black duck *(Anas rubripes*, hereafter black duck) and lesser scaup *(Aythya affinis)* are exhibiting decreasing long-term population trends. Therefore, they have generated significant conservation interest from the waterfowl management and research community (Baldassarre 2014a; U.S. Fish and Wildlife Service ; Ringelman and Williams 2018; U.S. Fish and Wildlife Service Migratory Bird Data Center 2019). Because of large-scale degradation and/or loss of historic coastal and inland wetlands (Dahl 1990, 2000, 2006), priority has been given to quantify the non-breeding season populations for these species (Austin et al. 2000; Devers and Collins 2011). To quantify the ability of a given landscape to support migrating and wintering waterfowl populations, bioenergetics modeling is commonly used to calculate energetic carrying capacity in terms of duck-use days by estimating energetic demand and energetic supply (Foster et al. 2010; Williams et al. 2014; Livolsi 2015; Lower Mississippi Valley Joint Venture 2015; Soulliere et al. 2017).

To estimate daily energy expenditures (DEE), researchers can build behavioral time-energy budgets and apply energetic equivalents for each behavior (Albright 1981; Cramer 2009; Jones et al. 2014; Livolsi 2015). Altough this approach requires substantial investments, it offers a thorough approach to estimating DEE while accounting for observed environmental variation as compared to Prince’s (1979) estimation that RMR x 3 would account for daily behavioral variability and environmental variation. Using the time-energy (*TE*) budget methodology, DEE is specifically estimated via:

$$DE{E_{TE}}=\mathop \sum \limits_{{hr=1}}^{n} ~\mathop \sum \limits_{{i=1}}^{n} ~\left[ {\left( {\left( {RMR \times {a_i}} \right)+CT} \right) \times {T_i}} \right]$$

where *RMR* = resting metabolic rate (kJ/bird/hr), *a*_*i*_ = activity specific factorial increase in *RMR* for the *i*th behavioral activity, *CT* = cost of thermoregulation at a specified temperature (kJ/bird/hr), *T*_*i*_ = proportion of time engaged in the *i*^*th*^ behavioral activity, and all values are summed across behaviors (*i*) and hours (*hr*) within the 24-hr cycle.

Species specific RMR has been studied for black ducks and estimates range between 309 and 523 kJ/bird/d (Hartung 1967; Berger et al. 1970; Wooley and Owen 1977; Bennett and Harvey 1987). Further, Miller and Eadie (2006) developed an equation based on regressing previously published estimates of RMR on body mass (RMR = *a* × Mass^*b*^), which enables researchers to produce an RMR estimate for any waterfowl species using input of only body mass and guild specific constants for mass proportionality coefficient (*a*) and slope of regression (*b*). Finally, the activity specific multipliers to RMR, *a*_*i*_, were estimated for black ducks by Wooley (1976) via surgically implanted heart rate monitors and found multiplier values of 1.7 for feeding, 1.2 for resting, 2.1 for comfort, 2.2 for swimming, 2.2 for alert, 12.5 for flying, and 1.7 for walking. Using these estimates, Albright et al. (1983) further estimated multipliers of 2.2 for agonistic and 2.4 for courtship behaviors. However, the accuracy of these estimates has been questioned (Williams et al. 2014) because Wooley (1976) explicitly acknowledged that wing-clipped, radio-equipped birds outfitted with heart rate monitors expressed marked behavioral difference as compared to those without them. Additionally, Albright’s (1983) multiplier estimates for agonistic and courtship behaviors appear to be approximations based on multiplier estimates of similar behaviors from Wooley (1976). Further, Goldstein (1988) pointed out the idiosyncratic relationship between heart rate and metabolic rate, suggesting that using the former to index the latter may not be appropriate. Lastly, Wooley (1976) performed observations starting in June and extended through summer, a timeframe that coincides with the waterfowl breeding season and generates concern that physiological changes associated with breeding could have impacted baseline energetic levels and, therefore, may not provide accurate behavioral multiplier estimates for the non-breeding season. Despite these concerns, multiplier estimates from Wooley (1976) and Albright et al. (1983) have consistently been used in many studies to estimate DEE in black ducks (Hickey 1980; Albright et al. 1983; Morton et al. 1989; Cramer 2009; Jones et al. 2014; Livolsi 2015) and to estimate DEE in other species (e.g., Atlantic brant [*Branta bernicla*] Ladin et al. 2011; Heise 2012).

RMR for lesser scaup and other diving species has been investigated as part of several research projects and estimates range between 233 and 405 kJ/bird/d (Stephenson 1994; McNab 2003; Kaseloo and Lovvorn 2005, 2006; Miller and Eadie 2006; Richman and Lovvorn 2011). Although activity specific multipliers to RMR for lesser scaup may, in some cases, be extrapolated from existing data (Kaseloo and Lovvorn 2005) for a subset of activities (resting, swimming, and preening), multipliers were not the intended output of these studies and methods of derivation varied between projects. Further, accurate estimates of DEE rely on RMR multiplier estimates for each of the full suite of activities observed during instantaneous scan samples.

If either estimates of activity specific multipliers to RMR or allometrically derived estimates of RMR are inaccurate or biased, the landscape level habitat management decisions being made based on duck-use day estimates could potentially be flawed. Because duck-use day and carrying capacity models scale individual energetic requirements to populations across large spatial extents, even small biases in RMR or behavioral multipliers can propagate to substantial error in estimates of habitat capacity and conservation targets (Williams et al. 2014; Pearse and Stafford 2014). The primary objective of this research is to produce estimates of activity specific multipliers and RMR for black ducks and lesser scaup using consistent and well-structured respirometry methods (Lighton 2008). This research fills important data gaps and refines energetic requirement estimates for black ducks and scaup. It is also anticipated that, because black ducks and lesser scaup represent common species from both the dabbling and diving duck guilds, this research may allow for reasonable extrapolation to additional species (i.e., mallards [*Anas platyrhynchos*], greater scaup [*Aythya marila*], redhead [*Aythya Americana*]).

## Methods

### Study area and experimental animals

We performed trials at the U.S. Geological Survey’s Patuxent Wildlife Research Center (PWRC) in Laurel, Maryland. Study black ducks (*n* = 6; 3 male and 3 female) and lesser scaup (*n* = 6; 3 male and 3 female) were hatched in captivity approximately May 2014 and pen-raised on-site at PWRC. When not involved in experimental trials, study ducks were housed and maintained outdoors among PWRC’s larger “duck colony” in mesh covered open-air enclosures (11.5 m^2^) with gravel substrates. Each enclosure contained an artificial conical rubber-lined pool (2.1 m diameter, 70–80 cm center depth) with constantly flowing fresh water. Except during fasting periods prescribed by individual research projects, ducks were fed (Mazuri Sea Duck Diet #5681, St. Louis, Missouri, USA) ad libitum and were provided crushed oyster shell during the breeding season. Research trials were performed in a plexiglass-fronted dive tank (1.82 m long × 0.91 m wide × 0.91 m water depth) inside a small research outbuilding (7.5 m x 10.5 m), which had independent climate and lighting control. The dive tank was outfitted with a continuous filtration system and was cleaned on an as-needed basis. All animal handling and research trial procedures followed PWRC Institutional Animal Care and Use Committee approved protocol (IACUC ID #: 2011-11 September 2013).

As ~ 4-month old ducklings, we placed study birds in the dive tank and a plexiglass respirometry chamber was lowered over the bird for short periods, weekly, in an attempt to minimize possible elevated metabolic rates associated with unfamiliar surroundings during actual respirometry trials. We monitored behaviors during these acclimation periods and variation in behavior (i.e., diving, feeding, etc.) was encouraged using food incentives. To ensure activity specific multiplier estimates for non-breeding ducks, and to avoid potential bias associated with physiological changes during the breeding season (Wooley and Owen 1977), we performed respirometry trials between October 2015 and March 2016. Trials occurred at air temperatures between 12 and 30 °C and water temperatures between 11 and 22 °C. Air and water temperature ranges were selected with the intent to maintain temperatures within the thermoneutral zone and to minimize effects of thermoregulation on energy expenditure (McKinney and McWilliams 2005). Air temperatures inside of the chamber and water temperatures at a depth of 5 cm were measured continuously at 1-s intervals during each trial. Study ducks were fasted overnight for at least 12 h prior to trials in order to ensure a post absorptive state. Prior to each trial, ducks were moved indoors to air-dry for 2 h before weighing as described in Lovvorn (1991) and Richman and Lovvorn (2008). We performed two trials per day, the first between 0930 and 1130 h (AM) and the second between 1230 and 1430 h (PM). We used a stratified randomization tool to develop a trial schedule which ensured that no single study bird underwent two trials in a single day and ensured that trials for each species were represented across the entire 6-mo study season.

### Respirometry

We used flow-through respirometry techniques adapted from Richman and Lovvorn (2008). Ambient air was drawn through a respirometry chamber via a mass flow meter (model FK-100, FlowKit Mass Flow Generator, Sable Systems International, Las Vegas, NV, USA) at a flow rate of 23 L/min. We selected this flow rate based on the specific configuration of our respirometry system and the average body masses of our study species (Lighton 2008). Sub-samples (250 mL/min) of excurrent air (already passed through the chamber) were passed through a multi-gas analyzer (model FMS Field Metabolic System, Sable Systems International, Las Vegas, NV, USA). The analyzer provided data outputs at 1-s intervals for each of eight distinct data channels including: chamber air temperature in °C, water temperature in °C, flow rate in mL/min, barometric pressure (BP) in kPa, water vapor pressure (WVP) in kPa and percent concentrations of oxygen (O_2_) and carbon dioxide (CO_2_). Drift in gas analysis was accounted for using periodic automated baselining during each trial where ambient air was periodically passed through the system to quantify and correct for analyzer drift (Lighton 2008). Specifically, our trials lasted 2 h each and began and ended with a 3-min baseline event with five additional 3-min baseline events distributed at equal 16.5-min intervals throughout the trial. We calibrated the multi-gas analyzer following standard respirometry procedures (Lighton 2008), including calibration of CO₂ and WVP sensors every 3 days and daily calibration of O₂ sensors using dried ambient air.

**Fig. 1:**
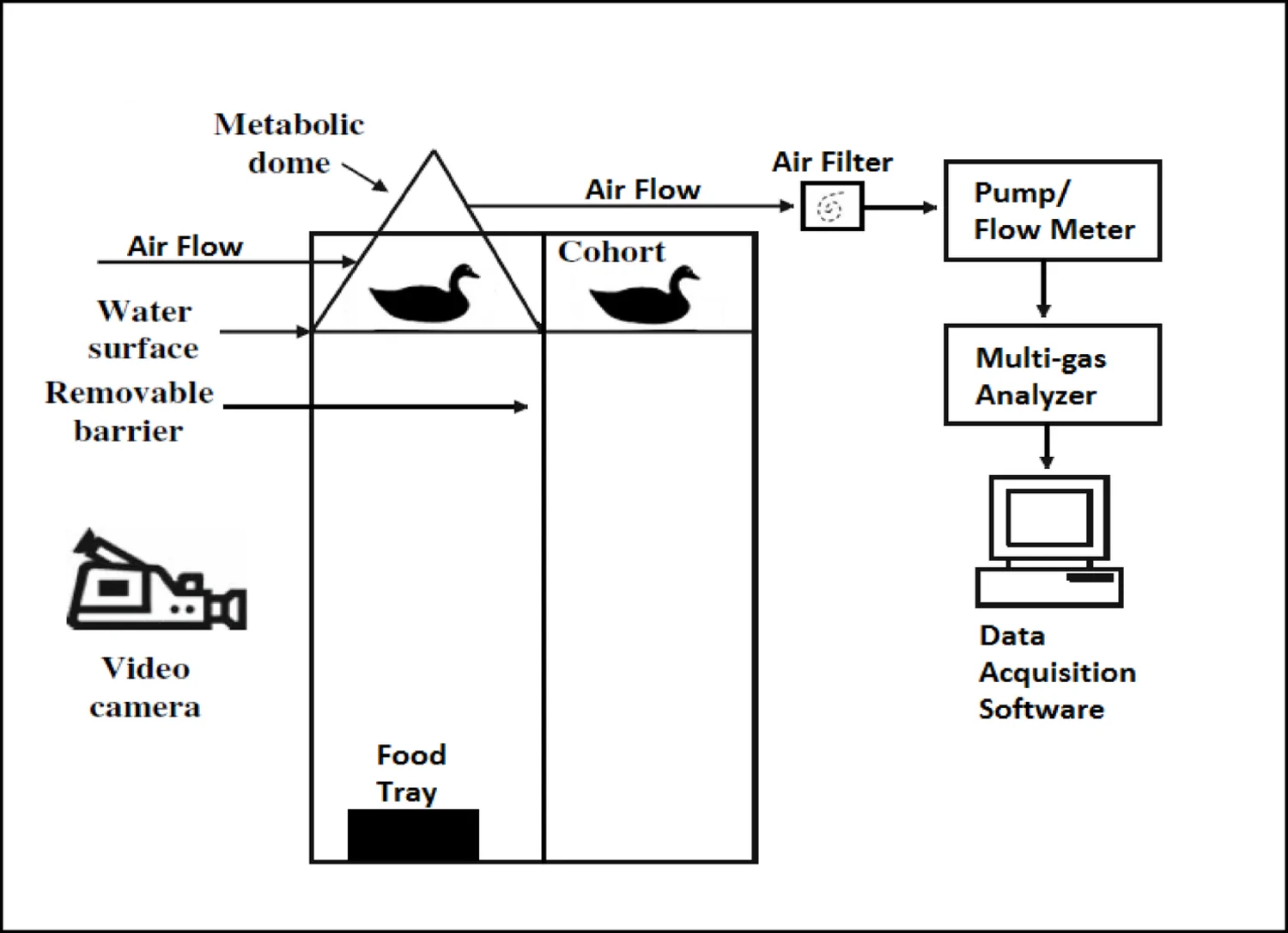
Apparatus used to estimate metabolic rates of captive American black duck (*n* = 6) and lesser scaup (*n* = 6) during respirometry trials at Patuxent Wildlife Research Center, Laurel, MD, October 2015–March 2016

During each trial, we placed a study duck on the water surface in the dive tank and lowered a truncated square pyramidal shaped plexiglass respirometry chamber around the duck until the base was submerged 5 cm to form an airtight seal with the water surface (Fig. 1). The chamber had a volume of 62.06 L (top width 33.66 cm, base width 53.98 cm, vertical height 31.75 cm) with incurrent air holes (1.2 cm dia. [× 4], positioned 5 cm from water surface) on one side and an excurrent hole (1.9-cm dia., positioned 15.9 cm from water surface) located on the opposite surface of the pyramid. A plastic mesh barrier was submerged in the tank to prevent study ducks from escaping the respirometry chamber underwater. During each trial, to reduce potential excessive anxiety and encourage interactive behaviors, either a live conspecific duck or a matched species decoy was placed in the dive tank in a mesh enclosure adjacent to the respirometry chamber. To encourage diving, dabbling, and feeding behaviors, we provided Mazuri Sea Duck Diet #5681 pellets and whole kernel corn either on the bottom of the tank (for scaup) or suspended 38 cm from the water surface (for black ducks) in 50% of trials (Fig. 1).

We videotaped trials, each of which lasted precisely 2 h. We performed a minimum of seven 2-hr trials for each study bird. In total, 90 trials were completed consisting of 45 trials for each species. Upon completion of trials, we used advanced data acquisition software (ExpeData, Sable Systems International, Las Vegas, NV, USA) to convert raw multi-gas analyzer data outputs (flow rate of excurrent air in L/min (FR), barometric pressure in kPa (BP), water vapor pressure in kPa (WVP), percent O_2_ in excurrent air, and percent CO_2_ in excurrent air) into estimates of $\:\dot{V}{o}_{2}$ (rate of O_2_ consumption [mL O2 s^-1^]) and $\:\dot{V}{co}_{2}\:$(rate of CO_2_ emission [mL CO2 s^-1^]) for the entirety of each trial (Lighton 2008).

### Behavioral analysis

Each 2-h respirometry trial was videotaped and time synchronized with the multi-gas analyzer output for retrospective analysis. From each trial video, we isolated individual behavior instances and behavior bouts. Specific behaviors of interest included: sleeping, resting, feeding (black ducks only), diving (lesser scaup only, includes feeding), comfort, swimming, agonistic, alert, and miscellaneous active (black ducks only). Descriptions of behaviors are detailed in Table S1; however importantly, sleeping behavior was classified as periods of complete inactivity characterized by absence of locomotion, minimal head or body movement, and sustained low rates of oxygen consumption relative to other observed behaviors (i.e., the lowest observed energy expenditure across behavioral states). These periods were used to operationally define resting metabolic rate (RMR) under controlled conditions.

Individual behavior instances were defined as a behavior of interest that a duck engaged in for a continuous period of *≥* 60 s. During acclimation trials (prior to actual experimental trials), we found that certain behaviors of interest were unlikely to meet the continuous 60-s threshold. In such circumstances, we analyzed behavior bouts, whereby average metabolic rates are estimated during a series or cycle of behaviors of interest (e.g., in the case of diving, estimating metabolic costs during a series of dives interspersed with one or more surfacing events; de Leeuw 1996). Therefore, behavior bouts were applicable only for the diving, feeding, and comfort behaviors and defined as a repeated occurrence of a single behavior of interest which, during a period of *≥* 60 s, included, at minimum, a single 30-s period where the duck was engaged in the behavior of interest and where any two instances of the target behavior were separated by no more than 15 s of rest. For each observed behavior or behavioral bout of interest, observers documented the precise time (seconds into the trial) that the behavior began and ended.

### Energetic expenditure

We used start and stop times for each recorded behavior to isolate the corresponding sections of multi-gas analyzer data outputs. Behavioral observations were time-synchronized with gas analyzer outputs, and although a short lag exists between chamber gas exchange and analyzer detection inherent to flow-through respirometry systems, this effect was minimized under continuous flow conditions and by restricting analyses to behavior segments of sufficient duration to ensure stable estimates of gas exchange (Lighton 2008). Using advanced data acquisition software (ExpeData, Sable Systems International, Las Vegas, NV, USA), we inserted distinct markers into the data output for each respirometry trial denoting the time periods that each study duck was engaged in a behavior or bout of interest. In general, we observed noticeably elevated $\:\dot{V}{o}_{2}$ and $\:\dot{V}{co}_{2}$ values at the beginning of each 2-h trial regardless of observed behaviors of interest. We assumed this was caused by human handling of study birds immediately prior to the start of each respirometry trial. To remove this energetic bias, we split each trial into consecutive 90-s intervals starting at the beginning of the trial and plotted the average percent change in $\:\dot{V}{o}_{2}$ and $\:\dot{V}{co}_{2}$ (mL/s) between each consecutive 90-s interval and identified where consecutive change was asymptotically < 1% (Odum and Keunzler 1955). This condition was met for both black ducks and lesser scaup at the 15th 90-s interval, and, thus, we deleted all behaviors between 0 and 1350 s.

For energetic analysis of behaviors that began or ended during a baseline event, we removed the portion of the behavior that occurred during the baseline (Lighton 2008) and still included the behavior instance in energetic analysis if, after removal of the baseline portion, it still met our required parameters (e.g., 60-s in length). For behaviors which extended through an entire baseline event, we used linear interpolation between the last data point prior to the baseline event and the first data point following the baseline event to generate reasonable estimates of data points across the 3-min baseline event.

For all behaviors of interest that met our criteria for useable data, we used ExpeData to record the mean values (collected at one-second intervals) of $\:\dot{V}{o}_{2}$ and $\:\dot{V}{co}_{2}$. We exported these values to Microsoft Excel and calculated the Respirometry Exchange Ratio’s (RER) for each individual behavior (Lighton et al. 1987):

$$RER=\dot {V}c{o_2} \div \dot {V}{o_2}$$

as well as estimated an oxyjoule equivalent (* Oxe*[mL^-1^]) where:

$$Oxe=\left[ {15.97+5.164\left( {RER} \right)} \right].$$

Finally, we produced estimates of energy expenditure for each behavior using:

$$J/s=~\dot {V}{o_2} \times Oxe$$

We then converted energy expenditure estimate from J/s to estimates of kJ/bird/hr and kJ/bird/d using the following equations:

$$\left( {J/s} \right) \times \left( {3600s/hr} \right)=J/hr$$

$$\left( {J/hr} \right) \times \left( {0.001kJ/J} \right)=kJ/hr~\left( {{\text{Hourly Energy Expenditure}},{\text{ HEE}}} \right)$$

$$\left( {kJ/hr} \right) \times \left( {24hr/d} \right)=kJ/d~\left( {{\text{Daily Energy Expenditure}},{\text{ DEE}}} \right)$$

### Statistical analysis

We first tested the normality of the HEE for each species-specific behavior using a Shapiro–Wilk test (Shapiro and Wilk 1965). To test whether energetic expenditure was different between behaviors for each species, we used parametric univariate ANOVA with Tukey’s post hoc test (Fisher 1958; Tukey 1977). Because some independent samples of a single behavior occurred multiple times on a sampling day, we blocked on sampling event (i.e., unique individual x day interaction) to account for possible pseudo-replication of behavioral data. If Tukey’s post hoc test identified behaviors that were not significant from one another, we examined if similar behaviors could be combined for further analysis to identify if covariates (time of day [AM/PM], presence of live cohort, presence of food, individual’s weight, air temperature, and water temperature) affected energy expenditure within behaviors or behavior groups. We constructed 12 a priori models and ran mixed effect general linear models (GLMs). All models were fit as linear mixed-effects models with individual bird identity and date included as random effects. Fixed effects included categorical predictors (time of day, presence of a live cohort, and presence of food) and continuous covariates (body mass, air temperature, and water temperature). We tested for collinearity between the six variables and any variable R *≥* 0.8 would not be included in the same model. Only water and air temperature were correlated (black ducks *R* = 0.95, lesser scaup *R* = 0.86). All a priori models were compared using Akaike Information Criterion corrected for small sample size (AICc, Burnham and Anderson 2002). If ΔAIC value was < 2, we conducted weighted model averaging to determine the beta values for the best predicted covariates. For model averaged parameter estimation, we used the “zero method” where a parameter estimate (and error) of zero is substituted into those models where the given parameter is absent thus reducing the effect sizes (and errors) of predictors that only appear in models with small model weights (particularly when the predictors have weak effects; Burnham and Anderson 2002; Lukacs et al. 2010; Grueber et al. 2011). Significant variables within the top ranked models were estimated using Wald Chi-square test (*α* = 0.05).

We note that sample size was limited to six individuals per species due to logistical constraints associated with flow-through respirometry. However, each individual contributed repeated measurements across multiple behaviors and conditions, allowing us to implement a repeated-measures modeling framework. This approach increases statistical power by leveraging within-individual variation and reduces bias associated with small sample sizes when estimating fixed effects (Zuur et al. 2009). Thus, although the number of individuals was modest, the total number of observations and the use of mixed-effects models provided improved inference for behavioral differences in energy expenditure.

## Results

### American black duck

In black ducks, HEE for each behavior was normally distributed (Shapiro-Wilk Test *P* > 0.093) except for the miscellaneous active behavior (*P* = 0.02). Across the eight behaviors, there was a difference among behaviors (*F*_7,139.3_ = 18.14, *P* < 0.01). Pairwise differences among behaviors identified using Tukey’s post hoc test are presented in Table S2. The results of the Tukey’s post hoc test (Table S3) suggested that behaviors could be lumped into homogeneous subset groups of “high-energy behaviors” including agonistic, comfort, miscellaneous active, and swimming; “medium-energy behaviors” including alert, feeding, and resting; and “low-energy behaviors” including sleeping (Table 1). To confirm this grouping of behaviors, we conducted a second univariate ANOVA (blocking on sampling event) testing the three behavioral categories (high, medium, and low) with Tukey’s post-hoc test. The three behavior-energy levels were significantly different from each other (*F*_2,127.6_ = 47.12, *P* < 0.01), and post hoc tests revealed all three groups were different from each other (*P* < 0.01). Thus, high-energy behaviors were estimated as 28.12 *±* 0.40 kJ/hr, medium-energy behaviors were estimated as 20.71 *±* 0.30 kJ/hr, and low-energy behavior (sleeping) was estimated as 14.89 + 0.42 kJ/hr (Table 1).

**Table 1:** Resting metabolic rate (RMR), behavioral metabolic rate, behavioral multipliers, and homogenous behavior groupings for all target behaviors in black ducks (*n* = 6) and lesser scaup (*n* = 6) during respirometry trials at Patuxent Wildlife Research Center, Laurel, MD, Oct. 2015–Mar. 2016

Species	Energy Group	Behavior	Instances	kJ/Bird/hr		kJ/Bird/d		Multiplier		
				$\:\stackrel{-}{x}$	SE	$\:\stackrel{-}{x}$	SE	$\:\stackrel{-}{x}$	SE	Wooley (1976)
American black duck	RMR	Sleeping	27	14.89	0.42	357.28	10.11	–	–	–
	Medium	Resting	210	20.66	0.30	495.82	7.14	1.39	0.02	1.20
		Alert	5	20.77	3.50	498.48	84.04	1.40	0.24	2.20
		Feeding	6	22.33	1.91	535.92	45.87	1.50	0.13	1.70
		Average	221	20.71	0.30	496.97	7.10	1.39	0.02	1.70
	High	Misc. Act.	93	27.56	0.51	661.47	12.15	1.85	0.03	-
		Swimming	3	27.92	0.99	670.16	23.77	1.88	0.07	2.20
		Agonistic	75	28.55	0.71	685.27	16.93	1.92	0.05	2.20
		Comfort	11	30.00	1.21	719.90	29.08	2.01	0.08	2.40^a^
		Average	182	28.12	0.40	674.95	9.54	1.89	0.03	2.27
Lesser scaup	RMR	Sleeping	67	15.89	0.41	381.43	9.72	–	–	–
	Low	Resting	116	17.45	0.36	418.72	8.74	1.10	0.02	–
	Medium	Agonistic	14	22.10	1.30	530.47	31.22	1.39	0.08	–
		Comfort	43	26.34	1.01	632.06	24.34	1.66	0.06	–
		Average	57	25.30	0.86	607.11	20.62	1.59	0.05	–
	High	Swimming	2	28.81	4.68	691.46	112.43	1.81	0.29	–
		Diving/Feeding	28	30.06	1.00	721.50	24.06	1.89	0.06	–
		Average	30	29.98	0.96	719.50	23.11	1.89	0.06	–

^a^Wooley (1976) provides individual multiplier estimates to RMR for comfort (2.1), preen (1.6), bath (2.9), and wing flap (3.0) behaviors. Our research lumped these behaviors into a single “comfort” category. Therefore, for compatibility, the “comfort” multiplier reported for Wooley (1976) is an average of the multiplier estimates for the previously mentioned behaviors

Resting metabolic rate (RMR) was estimated using measurements of sleeping behavior (identified as periods of low-energy consumption). We expanded our average sleeping HEE estimate to a DEE of 357.28 kJ/bird/d. Using Miller and Eadie (2006) estimation of RMR where *RMR = a* × *Mass*^*b*^ and where *a* represents a mass-proportionality coefficient, Mass represents body mass (kg), and *b* represents the slope of the regression line on a log scale (we used the values for a and b reported for “dabbling ducks” *a* = 457, *b* = 0.77). We used the daily weight of each of the ducks from this study before a trial and estimated RMR only for trials in which sleeping was recorded. Removing pseudo-replication, there were seven unique sampling events where sleeping energy consumption was estimated. The Miller and Eadie (2006) model estimated our birds’ RMR was 586.82 ± 18.65 kJ/bird/d which was significantly higher than our estimate of 357.28 *±* 10.11 kJ/bird/d (paired t test, *t*_6_ = 12.31, *P* < 0.01). Wooley and Owen (1977) estimated an average value of RMR for two birds at 327.80 kJ/bird/d, which was similar to our value of 357.28 kJ/bird/d (one-sample t test *t*_6_ = 1.04, *P* = 0.34).

We estimated energetic multipliers to RMR for each behavior and compared them against Wooley (1976) and Albright et al. (1983; agonistic behavior only). Behavioral multipliers were grouped into high- and medium-energy categories, with high-energy behaviors averaging 1.89 ± 0.03 × RMR and medium-energy behaviors averaging 1.39 ± 0.02 × RMR. Detailed estimates for each behavior and comparisons with Wooley (1976) are presented in Table 1.

**Fig. 2:**
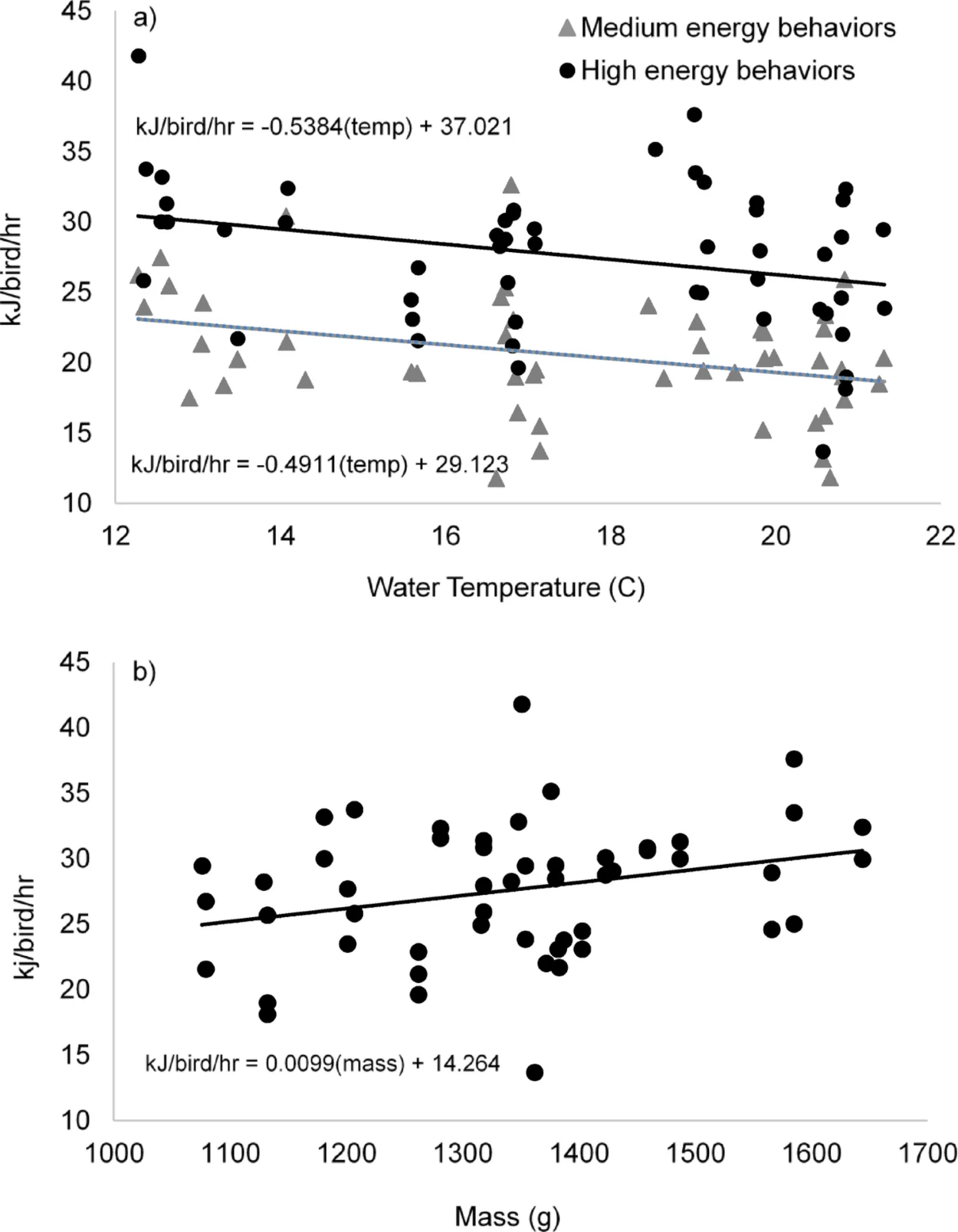
Effects of **a** water temperature on medium-energy behaviors and high-energy behaviors and **b** mass on high-energy behaviors expressed by American black ducks during respirometry trials at Patuxent Wildlife Research Center, Laurel, MD, October 2015–March 2016

Testing covariate effects against low-energy expenditure (RMR, sleeping behavior), the Null model (model weight = 0.74) best predicted energy expenditure and, thus, only the intercept (14.52 kJ/bird/hr) was significant (*χ*^*2*^ = 359.40, *P* < 0.01, Table S3). Testing covariate effects on medium-energy expenditure behaviors, two plausible models existed (combined model weight = 0.57) including “water temperature” and “weight + water temperature”. Thus, the following model best predicted energy expenditure:

$$\begin{aligned} & Medium - energy{\text{ }}expenditure{\text{ }}\left( {kJ/bird/hr} \right) \\ & \quad =26.205+0.002{\text{ }}\left( {weight} \right) \hfill \\ & \qquad -0.490{\text{ }}\left( {water{\text{ }}temperature} \right) \hfill \\ \end{aligned} $$

and the intercept and water temperature showed significance (intercept: *χ*^*2*^ > 12.95, *P* < 0.01; water temperature: *P* < 0.02, weight: *P* = 0.16; Table S3, Fig. 2). Testing covariate effects on high-energy expenditure behaviors the following model (model weight = 0.41) best predicted energy expenditure:

$$\begin{aligned} & High - energy{\text{ }}expenditure{\text{ }}\left( {kJ/bird/hr} \right) \\ & \quad =23.780+0.010{\text{ }}\left( {weight} \right) \hfill \\ & \qquad -0.579{\text{ }}\left( {water{\text{ }}temperature} \right) \hfill \\ \end{aligned} $$

and all variables showed significance (intercept: *P* < 0.01; weight: *P* = 0.02, water temperature: *P* = 0.01; Table S3, Fig. 2).

### Lesser scaup

In lesser scaup, HEE estimates for each behavior were normally distributed (Shapiro-Wilk Test, *P* ≤ 0.05). Across the six behaviors, there were differences between behaviors (univariate ANOVA with Tukey’s post hoc test, *F*_5,101.02_ = 25.63, *P* < 0.01). Pairwise differences among behaviors identified using Tukey’s post hoc test are presented in Table S2. Like black ducks, results of the Tukey’s post hoc test suggested that certain behaviors with similar energetic expenditures could be broken into homogeneous subset groups. However, lesser scaup included four behavior energy groups as opposed to three in black ducks. Lesser scaup behavior energy groups included “high-energy behaviors” including diving and swimming behaviors, “medium-energy behaviors” including agonistic and comfort behaviors, “low-energy behaviors” including resting, and “extreme low-energy behaviors” (e.g., RMR) including sleeping (Table 1). Once again, to confirm this lumping of behavioral groups, we conducted a second univariate ANOVA (blocking on sampling event) testing the four behavioral categories (high, medium, low, and RMR) with Tukey’s post-hoc test. The four behavior-energy levels were significantly different from each other (*F*_3,113.81_ = 45.55, *P* < 0.01) and post hoc tests revealed all four groups were different from each other (*P* < 0.02). Thus, high-energy behaviors were estimated as 29.98 + 0.96 kJ/hr, medium-energy behaviors were estimated as 25.30 ± 0.86 kJ/hr, low-energy behavior was estimated as 17.45 ± 0.36 kJ/hr, and RMR was estimated as 15.89 ± 0.41 kJ/hr (Table 1).

As with black ducks, we considered estimates of sleeping energy expenditure in lesser scaup to be a reasonable estimate of resting metabolic rate due to our methodological design. We expanded the average sleeping HEE estimate to DEE of 381.43 kJ/bird/d (Table 1). Using Miller and Eadie’s (2006) allometric estimation of RMR with values for *a* and *b* reported for “diving ducks” (*a* = 446, *b* = 0.98) and the daily weight of each study duck before a trial, we estimated RMR only for trials in which sleeping was recorded. Removing pseudo-replication, there were 13 unique sampling events where sleeping energy consumption was estimated. The Miller and Eadie (2006) model estimated RMR for our study birds as 354.84 *±* 11.33 kJ/bird/d which was not statistically different from our estimate of 381.43 *±* 9.72 (paired t-test, *t*_10_=1.00, *P* = 0.34).

We estimated energetic multipliers to RMR for each behavior and grouped them into high-, medium-, and low-energy categories. High-energy behaviors averaged 1.89 ± 0.06 × RMR. This pooled estimate was calculated from the combined high-energy HEE data and, because most observations were diving/feeding (*n* = 28) rather than swimming (*n* = 2), it rounded to the same value as diving/feeding alone. Medium-energy behaviors averaged 1.59 ± 0.05 × RMR, and low-energy behavior (resting) averaged 1.10 ± 0.02 × RMR. Detailed estimates for each behavior are presented in Table 1. Testing covariate effects against RMR energy expenditure (sleeping), the null model (model weight = 0.61) best predicted energy expenditure and thus only the intercept (15.766 kJ/bird/hr) was significant (*P* < 0.01, Table S4). Testing covariate effects on low-energy expenditure behavior (resting), four models were plausible (combined weight = 0.64) including “null”, “water temperature”, “air temperature”, and “time of day”. Thus, using the “zero-method” of parameter model averaging, the final model best predicted energy expenditure:

$$\begin{aligned} & Low - energy{\text{ }}expenditure{\text{ }}\left( {kJ/bird/hr} \right) \\ & \quad =19.443 - 0.281{\text{ }}\left( {PM} \right) \hfill \\ & \qquad -0.051{\text{ }}\left( {air{\text{ }}temperature} \right) - 0.076{\text{ }}\left( {water{\text{ }}temp} \right). \hfill \\ \end{aligned} $$

In all four models, the intercept was significant (*P* < 0.01), however the other three variables were not significant (*P* > 0.13, Table S4). Testing covariate effects on medium-energy expenditure behaviors (agonistic and comfort), two models were plausible (combined weight = 0.71) including “time of day” and “time of day + food presence”. Thus, using the “zero-method” of parameter model averaging, the final model best predicted energy expenditure:

$$\begin{aligned} & Medium - energy{\text{ }}expenditure{\text{ }}\left( {kJ/bird/hr} \right) \\ & \quad =21.825+6.257{\text{ }}\left( {AM} \right) \hfill \\ & \qquad +0.779{\text{ }}\left( {food{\text{ }}present} \right). \hfill \\ \end{aligned} $$

**Fig. 3:**
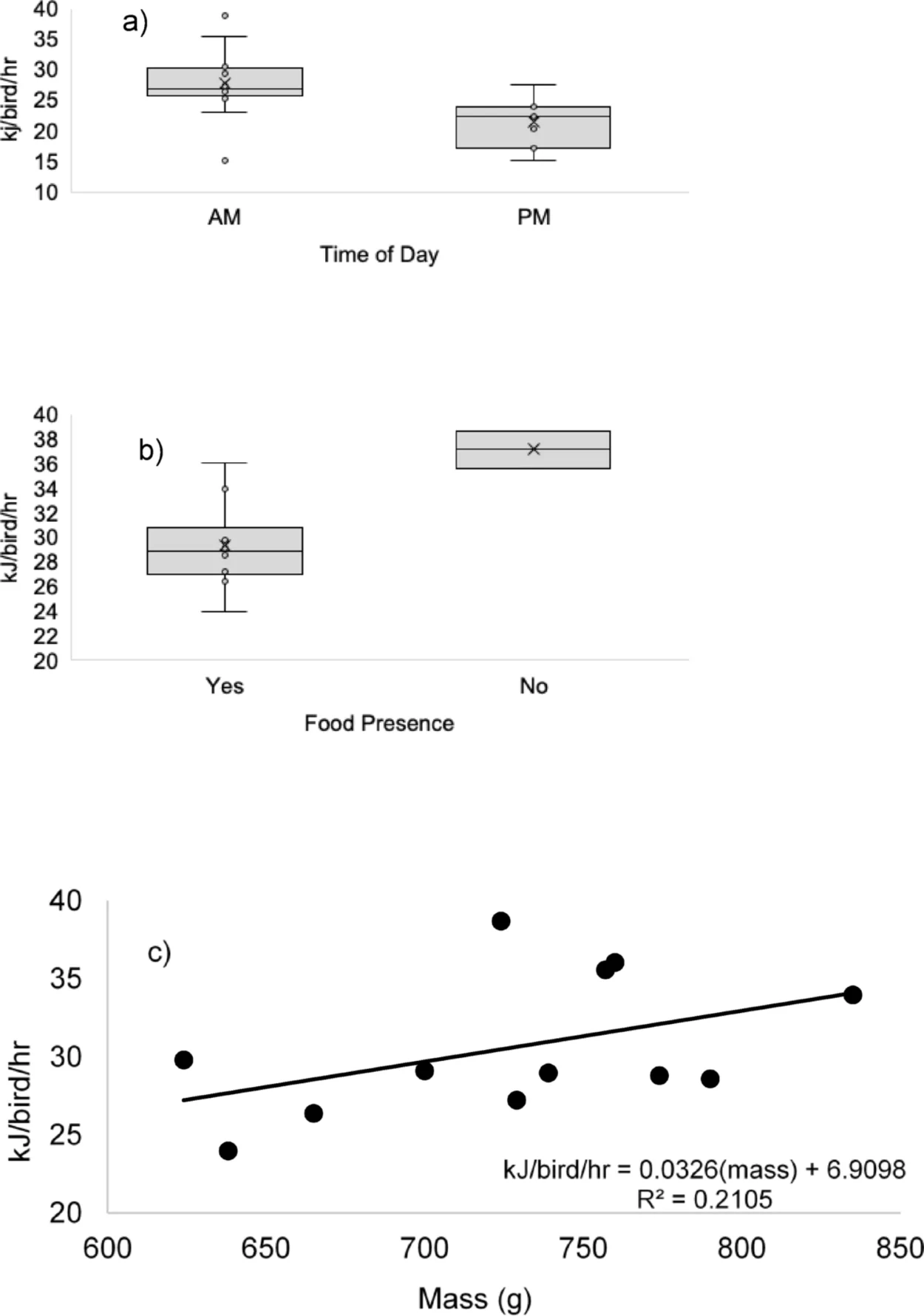
Effects of **a** time of day on medium-energy behaviors, **b** food presence on high-energy behaviors, and **c** bird mass on high-energy behaviors expressed by lesser scaup during respirometry trials at Patuxent Wildlife Research Center, Laurel, MD, October 2015–March 2016

In both models, the intercept was significant (*P* < 0.01) and time of day was significant in both models with morning energy expenditure being 6.26 kJ/bird/hr higher than afternoon expenditure (*P* < 0.01), however presence of food in tank was not significant (*P* = 0.23, Table S4, Fig. 3). Testing covariate effects on high-energy expenditure behaviors (swimming and diving), two models were plausible (combined weight = 0.66) including “food presence”, and “food presence + weight”. Thus, using the “zero-method” of parameter model averaging, the final model best predicted energy expenditure:

$$\begin{aligned} & High - energy{\text{ }}expenditure{\text{ }}\left( {kJ/bird/hr} \right) \\ & \quad =20.273 - 7.661{\text{ }}\left( {food{\text{ }}present{\text{ }}in{\text{ }}\tan k} \right) \hfill \\ & \qquad +0.012{\text{ }}\left( {weight} \right). \hfill \\ \end{aligned} $$

In one of the two models, the intercept was significant (*P* < 0.01) and each of the other variables were significant in their single models (food presence: *P* = 0.01, weight: *P* < 0.01, Table S3, Fig. 3).

## Discussion

A primary impetus for this research was the limited availability of studies investigating behavioral multipliers to RMR in waterfowl. We used updated, replicable respirometry techniques to estimate behavioral multipliers and RMR for black ducks and lesser scaup. We also evaluated the accuracy of behavioral multiplier estimates derived from past studies that relied on more invasive and potentially biased methods. Estimates from Wooley (1976) have been used extensively to model DEE and landscape carrying capacity across waterfowl species and regions (Hickey 1980; Albright et al. 1983; Morton et al. 1989; Cramer 2009; Ladin et al. 2011; Heise 2012; Jones et al. 2014; Livolsi 2015).

Previously published RMR estimates range from 309 to 523 kJ/bird/d for black ducks (Hartung 1967; Berger et al. 1970; Wooley and Owen 1977; Bennett and Harvey 1987; Miller and Eadie 2006) and 233–405 kJ/bird/d for lesser scaup (Stephenson 1994; McNab 2003; Kaseloo and Lovvorn 2005, 2006; Miller and Eadie 2006; Richman and Lovvorn 2011). Our RMR estimates for both species fell within these published ranges. Our estimate for lesser scaup did not differ significantly from allometric estimates derived using Miller and Eadie’s (2006) “diving duck” model. In contrast, our black duck RMR estimate was substantially lower than the corresponding allometric estimate. Notably, this allometric estimate falls outside the range of previously published values for black ducks.

Miller and Eadie’s (2006) allometric approach allows estimation of RMR and DEE using only body mass data. Although this approach is efficient, it may bias estimates of DEE and landscape carrying capacity. For example, Cramer (2009) used an allometric “dabbling duck” RMR estimate (514 kJ/bird/d), which exceeded most published black duck RMR values, to derive a DEE estimate (1,187 kJ/bird/d) that was higher than previously reported values (664–745 kJ/bird/d [Hickey 1980; Albright et al. 1983; Morton et al. 1989 ]).Subsequent studies using similar allometric RMR estimates (e.g., Jones et al. 2014; Livolsi 2015) likewise produced DEE values that exceeded previously published estimates, suggesting that elevated RMR inputs contributed to these discrepancies. To evaluate the implications of our revised estimates, we substituted our RMR and grouped behavioral multipliers into Cramer’s (2009) modeling framework. This produced a DEE estimate of 802 kJ/bird/d; a 32.4% (385 kJ/bird/d) reduction, which could substantially alter landscape-level carrying capacity estimates.

These observations are not intended to diminish the utility of allometric equations as a practical tool for estimating RMR, DEE, and landscape carrying capacity. Allometric equations provide an efficient tool for researchers and managers, and their utility can continue to improve as species-specific RMR estimates are incorporated into updated models. Nevertheless, accuracy may improve when species-specific, experimentally derived estimates of RMR are available. This is especially true when allometric guild estimates fall near the fringes or outside the range of published species-specific values, as observed for black ducks. In contrast, allometric estimates may be appropriate for lesser scaup, where they fall within the range of published RMR values and may facilitate standardized approaches across studies. For black ducks, however, the relatively high allometrically derived “dabbling duck” RMR estimate may contribute to recurring overestimates of DEE and bias landscape carrying capacity estimates.

Although more time-consuming and costly, species-specific time-energy budget approaches allow researchers to refine DEE estimates by incorporating behavioral and environmental factors that are difficult to capture using allometrically derived RMR and a standard multiplier of three times RMR (Prince 1979). However, improvements in DEE estimates from time-energy budgets depend on the accuracy of underlying inputs, including RMR, activity budgets, behavioral multipliers, and thermoregulatory costs. Despite their importance, few recent studies have directly estimated behavioral multipliers to RMR, a key component of time-energy budget models. As a result, many studies (Hickey 1980; Albright et al. 1983; Cramer 2009; Heise 2012; Jones et al. 2014; Livolsi 2015) have relied on Wooley (1976), who estimated multipliers for black ducks during Maine summers. This reliance reflects the limited availability of empirically derived behavioral multipliers for waterfowl.

Our behavioral multiplier estimates for black ducks differed from those reported by Wooley (1976). With the exception of resting behavior, multipliers across all measured behaviors were lower than those published by Wooley (1976). We suggest that estimates derived from modern respirometry during winter provide more appropriate multipliers for estimating wintering DEE and landscape carrying capacities for black ducks. Although Wooley’s (1976) multipliers have been broadly applied across species, our estimates may be more appropriate for closely related dabbling ducks (e.g., mallards) until species-specific multipliers are developed.

In addition to black ducks, we produced behavioral multipliers estimates for lesser scaup. These estimates differed from those for black ducks and help fill a gap in the existing literature. Most waterfowl bioenergetics modeling has focused on dabbling ducks, likely because landscape energy availability for dabbling ducks in agricultural fields and managed impoundments has been a priority for habitat and harvest management. Our lesser scaup multiplier estimates therefore provide an opportunity to expand bioenergetics research focused on scaup and other diving species.

We do acknowledge that our results have limitations. Traditional time-energy budgeting applies behavioral multipliers across a range of behaviors including feeding, resting, comfort, swimming, alert, walking, agonistic, courtship, diving, and flight (Prince 1979; Wooley 1976). Although we estimated multipliers for most behaviors, our design did not allow estimation for courtship, flight, and walking. However, our results indicate that behaviors with similar energetic costs can be grouped and represented by a single behavioral multiplier when estimating DEE.

In black ducks, resting, feeding, and alert behaviors can be treated as medium-energy behaviors with a common multiplier of 1.39. Similarly, swimming, agonistic, and comfort behaviors can be treated as high-energy behaviors with a common multiplier of 1.89. Based on these groupings, walking and courtship behaviors may also reasonably be included in the high-energy category with a multiplier of 1.89 in future studies of black duck DEE. This interpretation is supported by our “miscellaneous active” category in black ducks, which included active behaviors outside our predefined categories or combinations of active behaviors that did not individually meet behavior thresholds (e.g., one behavior sustained for > 60 s). Thus, this category functioned as a catch-all for a range of active behaviors. 

For lesser scaup, agonistic and comfort behaviors can be treated as medium-energy behaviors with a common multiplier of 1.59. Swimming and diving/dive-feeding can be treated as high-energy behaviors with a common multiplier of 1.89. Following the same logic used with black ducks, walking and courtship behaviors may also reasonably be included in the high-energy category with a multiplier of 1.89 in future studies of lesser scaup DEE. Because flight is the most energetically costly behavior (Wooley and Owen 1978) and does not fit intuitively within our behavior groupings, we recommend continued use of Wooley and Owen’s (1978) multiplier estimate of 12.5 until future research refines species- and guild-specific energetics.

Many environmental and physiological factors affect energy expenditure in waterfowl. For the diving behavior alone, body size, body fat, dive depth/pressure, and water temperature affect energetic expenditure (Lovvorn and Jones 1991; Richman and Lovvorn 2011). Because our goal was to produce broadly applicable estimates, we controlled some factors (e.g., air and water temperature) but did not explicitly evaluate others. For covariates with available data, we conducted basic analysis to evaluate their effects on behavioral energy groupings, but these relationships could be studied further. No single covariate affected energy expenditure across both species and behavior groupings. This finding supports analyzing covariate effects within grouped behaviors rather than across all behaviors and suggests that some factors influence energy expenditure differently among behaviors. Covariates did not affect energy expenditure during low-energy sleeping behavior for either species. This further supports the use of average sleeping energy expenditure as an estimate of RMR and as a basis for behavioral multipliers in each species.

Experimental conditions within the thermal neutral zone (> 12° C; Wooley and Owen 1977) support the use of sleeping behavior as a proxy for resting metabolic rate under controlled conditions. These estimates are not intended to represent strict basal metabolic rate (BMR), which is typically defined under more restrictive conditions (e.g., post-absorptive state, minimal activity, and standardized environmental conditions), but rather reflect RMR as operationally defined in this study based on periods of inactivity and minimal energy expenditure under controlled conditions. Differences among published estimates of metabolic rate may therefore reflect variation in definitions and experimental protocols.

In black ducks, water temperature did not significantly predict low-energy sleeping behavior energy (RMR), as expected under these thermal neutral conditions. However, energetic demand in medium- and high-energy behaviors increased as water temperature decreased from ~ 21° C to ~ 12° C, and this effect was greater for higher energy behaviors (Fig. 2). In lesser scaup, water temperature did not significantly predict energy expenditure for any behavior. Richman and Lovvorn (2011) showed that water temperature can strongly influence thermoregulation costs in aquatic birds and that past DEE estimates may underestimate these costs. De Leeuw (1996) likewise showed that the effect of water temperature on diving energetics may depend on the measurement approach. This may help explain the absence of water temperature effects in lesser scaup. Despite evidence that water temperature affects energy expenditure (McKinney and McWilliams 2005), most waterfowl DEE studies emphasize only air temperature and wind speed in thermoregulatory costs. This relationship could be examined more thoroughly and incorporated into future DEE calculations.

It is widely acknowledged that body mass affects energy expenditure (McKinney and McWilliams 2005; Miller and Eadie 2006). We found that energy expenditure increased with mass in both black ducks and lesser scaup engaged in high-energy behaviors (swimming, agonistic, comfort, and diving). In black ducks, high-energy behavior expenditure increased by 0.99 kJ/bird/hr for every 100-g increase in body mass. Similarly, lesser scaup exhibited a 3.26 kJ/bird/hr increase in high-energy behavior expenditure for every 100-g increase in body mass. This supports previous findings and suggests that the relationship between body mass and energy expenditure is most apparent during energetically costly behaviors.

Time of day influenced energy expenditure in lesser scaup engaged in medium-energy behaviors (agonistic and comfort), with individuals expending more energy in the morning than in the afternoon. Previous time-activity budget studies have also documented diel variation in energy expenditure (Jones et al. 2014; Livolsi 2015), but those patterns are typically attributed to shifts in the proportion of time allocated to different behaviors. In contrast, our analysis compared energy expenditure within similar behaviors and therefore isolates temporal differences independent of activity budgets. This suggests that the observed pattern reflects an inherent difference in energetic cost between morning and afternoon. One possible explanation is that physiological or behavioral state early in the day may elevate energetic demand, although this was not explicitly tested. Variation in behavior duration was not incorporated into our models and may also contribute to this pattern. Contrary to expectations, lesser scaup engaged in high-energy behaviors (swimming and diving) expended more energy when food was absent. This pattern may reflect increased search effort or more sustained foraging-related activity when food was not immediately available, which could elevate energetic expenditure during these behaviors. However, because we did not explicitly quantify search intensity or fine-scale behavioral responses to food availability, this mechanism remains speculative. We also note that aspects of our methodological design may have contributed to this pattern. Given these uncertainties, we caution against attributing strong conservation implications to this finding. 

In hindsight, several methodological improvements could be made, and some unavoidable limitations should be noted. Lighton (2008) emphasized the importance of anticipated O_2_ depletion when designing flow-through respirometry systems. This consideration is critical for both animal safety and data quality. For example, estimating metabolic rates of a large animal in a small chamber with low flow rates may expose the animal to hypoxia and/or hypercapnia. Conversely, a small animal in a large chamber with high flow rates may yield inaccurate estimates due to limited detection of respiration-driven gas changes. Because this study measured metabolic rates across behaviors, including active behaviors, we had to balance gas exchange constraints with the ability of ducks to perform those behaviors. Our chamber design allowed ducks to engage in defined behaviors, but confinement likely influenced behavior type and duration relative to free-ranging conditions. We were also unable to measure flight energetics, which is the most energetically costly behavior (Wooley and Owen 1978). Future work could investigate flight energetics using wind tunnels and doubly labeled water (Masman and Klaassen 1987).

When prescribed, a fixed amount of food was placed in the dive tank at the beginning of a trial, and birds were allowed to feed at will. Because birds were fasted, they typically began feeding immediately upon entering the chamber and consumed available food before the threshold for usable behaviors (1,350 s after trial start). Although elevated energy expenditure early in trials may reflect rapid feeding, this pattern occurred in approximately 50% of trials without food, suggesting it was not driven solely by feeding or handling. If handling effects were the primary driver, we would expect more feeding behavior after food introduction in both species. Future studies could consider introducing food at regular intervals throughout trials.

There is extensive research on the energetic cost of diving in ducks. However, few studies provide multipliers to RMR for this behavior. In many cases, multipliers can be derived from reported data (Woakes and Butler 1983; Daan et al. 1990; Bevan and Butler 1992; Stephenson 1994; de Leeuw 1996). Because our study estimated energetic expenditure estimates across a broad range of behaviors, many of the considerations typical of diving-specific studies were beyond our scope. Nonetheless, our estimate of average diving energy expenditure (721.50 SE = 24.06 kJ/bird^/^d) fell within, but on the low end of, previously reported values (691.2–1,270.1 kJ/bird/d [Woakes and Butler 1983; Daan et al. 1990; Bevan and Butler 1992; Stephenson 1994; de Leeuw 1996 ]).

De Leeuw (1996) identified three approaches to measuring diving energetics: 1) metabolic rates during submergence, 2) metabolic rates during dive and breathing intervals (bouts), and 3) metabolic rates over a series of dives including post-dive recovery. The third approach provides the most comprehensive estimate and revealed covariate effects, including an inverse relationship between diving costs and water temperature, that were not apparent using the second approach. Our design required use of the second approach. Although evaluating covariate effects was not a primary objective, we similarly found no relationship between water temperature and diving costs using this approach. Future studies examining diving energetics or covariate effects could consider these methodological differences.

Body size, body fat, dive depth/pressure, air temperature, and water temperature have been shown to affect the energetic cost of diving (Lovvorn and Jones 1991; Richman and Lovvorn 2011). Because our goal was to produce broadly applicable multiplier estimates, we did not specifically investigate all these relationships. Our dive tank limited dives to approximately 1 m, which likely contributed to our estimate falling on the low end of previously published values and may underestimate costs across the full range of dive depths experienced by free-living ducks. Lesser scaup in the wild dive to depths of up to 12 m to acquire food (Baldassarre 2014b).

We acknowledge that air and water temperatures under captive conditions were narrower and generally warmer than those encountered by wild wintering birds. This limited range likely reduced our ability to detect temperature-dependent relationships and may explain the absence of significant effects in some models, despite previous work showing strong temperature dependence (Bevan and Butler 1992; Richman and Lovvorn 2011). Consequently, absolute energy expenditure estimates are likely conservative relative to free-living birds in colder environments. Because trials were conducted within the thermal neutral zone, our estimates represent baseline energetic costs without substantial thermoregulatory demand. Under field conditions outside this range, additional thermoregulatory costs should be incorporated into DEE estimates through the CT term. In some cases, activity-generated heat may partially offset thermoregulatory demands, although this relationship is context-dependent. Application of these results to free-living populations should account for temperature-dependent thermoregulatory costs.

In addition, because this study used captive, pen-raised individuals, metabolic rates may be lower than those of free-living birds, as documented in other avian systems (McKechnie et al. 2006). Captive birds may experience reduced energetic demands due to limited movement, absence of predation risk, and controlled environmental conditions. As a result, absolute estimates of RMR and DEE may be conservative when extrapolated to wild populations. This potential bias should be considered when applying these values to landscape-scale carrying capacity models, as underestimation of individual energetic demand could lead to overestimation of habitat capacity. However, because behavioral multipliers are expressed relative to RMR, they are less sensitive to this bias and remain broadly applicable for modeling relative energetic costs across behaviors.

Successful waterfowl conservation depends on accurately linking population objectives to habitat goals. For example, the Atlantic Coast Joint Venture established an objective of increasing the wintering population index of black ducks to 385,000 (ACJV 2005). Estimating the habitat required to meet such objectives depends on accurate estimates of landscape carrying capacity. In systems where food resources are limiting, carrying capacity reflects the balance between energetic demand and available resources. Previous work has developed methods to estimate carrying capacity (Williams et al. 2014), but their accuracy depends on underlying parameters (i.e., habitat composition, true metabolizable energy, RMR, behavioral multipliers, time-energy budgets, costs of thermoregulation). Because these parameters vary across seasons, regions, species, and individuals, exact estimates are not possible. Instead, researchers must rely on accumulated evidence to generate average parameter estimates that approximate population-level energetics. The parameter estimates from this research can be applied to improve wintering landscape carrying capacity estimates. For example, Fino et al. (2017) found that 584,988-ha of forested wetlands within 24.1 km of the Mid-Atlantic Coast provided approximately 11,286 kcal/ha of energy available to wintering black ducks. Using Cramer’s (2009) DEE estimate of 1,187 kJ/bird/d (283.7 kcal/bird/d), we would expect 1 ha of this habitat to support 40 black ducks for a single day. Using our refined DEE estimate of 802 kJ/bird/d (191.7 kcal/bird/d [this study, developed using Cramer 2009 time-energy budgets and cost of thermoregulation]), we expect this same hectare to support 59 black ducks for a day. Scaled to the landscape level, as compared to Cramer’s (2009) DEE estimate, our findings suggest that forested wetland habitats in this landscape can support over 11 million more duck-use-days. Thus, the refined energetics values estimated in this research can be used to improve future waterfowl habitat conservation planning and objectives.

## Supplementary Information

Below is the link to the electronic supplementary material.

Supplementary Tables

## Data Availability

The data supporting the findings of this study are available from the corresponding author upon reasonable request.
