# Supplementary material for: Estimating behavioral multipliers to American black duck and lesser scaup resting metabolic rate to better estimate daily energy expenditure
Source: J Comp Physiol B. 2026 Jun 22;196(4):563–77. doi: 10.1007/s00360-026-01672-1 (PMC13506555; doi:10.1007/s00360-026-01672-1)
Supplement: Supplementary file 1 [file 360_2026_1672_MOESM1_ESM.docx]

Estimating behavioral multipliers to American black duck and lesser scaup resting metabolic rate to better estimate daily energy expenditure

JACOB W. McPHERSON, *University of Delaware, 250 Townsend Hall, Newark, DE 19716, USA*

CHRISTOPHER K. WILLIAMS, *University of Delaware, 250 Townsend Hall, Newark, DE 19716, USA*

ALICIA M. BERLIN, *U.S. Geological Survey, Patuxent Wildlife Research Center, 12100 Beech Forest Road, Laurel, MD, 20708*

JOHN M. COLUCCY, *Ducks Unlimited Incorporated, 7322 Newman Boulevard, Building 1, Dexter, MI 48130, USA*

Table S1 - Descriptions of target behaviors for captive black ducks (*n* = 6) and lesser scaup (*n* = 6) during respirometry trials at Patuxent Wildlife Research Center, Laurel, MD, October 2015–March 2016.

| Behavior | Description |
| --- | --- |
| Sleeping | Eyes closed and/or head tucked |
| Resting | Head not tucked; eyes open but bird is not propelling itself or is otherwise inactive |
| Feeding | Black ducks only; tipped up subsurface feeding or obvious surface feeding of pelletized food; can occur as a behavior bout |
| Diving | Lesser scaup only; study bird is submerged underwater entirely; can include feeding; can occur as a behavior bout |
| Comfort | Includes splashing, bathing, preening, wing-flap, scratching, or other similar behaviors; can occur as behavior bout |
| Swimming | Bird is actively propelling itself using its feet |
| Agonistic | Apparent aggressive attempt at physical interaction with cohort bird |
| Alert | Head fully extended; bird not otherwise active |
| Misc. Active | Black ducks only; bird is active, but behavior does not fit into any of the above defined behaviors |

Table S2. Pairwise comparisons of behavioral hourly energy expenditure (HEE) among behaviors for American black ducks and lesser scaup based on Tukey’s post hoc tests. during respirometry trials at Patuxent Wildlife Research Center, Laurel, MD, October 2015–March 2016. “ns” indicates non-significant differences (P > 0.05).

(A) American black duck

| Behavior | Sleeping | Resting | Feeding | Alert | Swimming | Comfort | Agonistic | Misc. Active |
| --- | --- | --- | --- | --- | --- | --- | --- | --- |
| Sleeping | – | <0.01 | <0.01 | <0.01 | <0.01 | <0.01 | <0.01 | <0.01 |
| Resting | – | – | ns (>0.87) | ns (>0.87) | <0.01 | <0.01 | <0.01 | <0.01 |
| Feeding | – | – | – | ns (>0.13) | ns (0.13) | <0.01 | <0.01 | <0.01 |
| Alert | – | – | – | – | <0.02 | <0.01 | <0.23 | <0.23 |
| Swimming | – | – | – | – | – | ns (>0.96) | ns (>0.96) | ns (>0.96) |
| Comfort | – | – | – | – | – | – | ns (>0.17) | ns (>0.17) |
| Agonistic | – | – | – | – | – | – | – | ns (>0.38) |
| Misc. Active | – | – | – | – | – | – | – | – |

(B) Lesser scaup

| Behavior | Sleeping | Resting | Agonistic | Comfort | Swimming | Diving |
| --- | --- | --- | --- | --- | --- | --- |
| Sleeping | – | 0.04 | <0.01 | <0.01 | <0.01 | <0.01 |
| Resting | – | – | <0.01 | <0.01 | <0.01 | <0.01 |
| Agonistic | – | – | – | <0.01 | ns (0.10) | <0.01 |
| Comfort | – | – | – | – | ns (0.92) | <0.01 |
| Swimming | – | – | – | – | – | ns (0.99) |
| Diving | – | – | – | – | – | – |

Table S3. Candidate models for estimating covariate effects on energy expenditure of black ducks (*n* = 6) engaged in low, medium, and high energy behaviors during respirometry trials at Patuxent Wildlife Research Center, Laurel, MD, October 2015–March 2016.

| Behavioral State | Model | k | AICc | Δ AIC | w |
| --- | --- | --- | --- | --- | --- |
| Low energy (RMR) | Null | 1 | 36.75 | 0 | 0.74 |
|  | Cohort Presence | 2 | 41.73 | 4.98 | 0.06 |
|  | Weight | 2 | 41.73 | 4.99 | 0.06 |
|  | Food Presence | 2 | 42.21 | 5.46 | 0.05 |
|  | Water Temp | 2 | 42.77 | 6.03 | 0.04 |
|  | Air Temp | 2 | 43.09 | 6.34 | 0.03 |
|  | Time of Day (AM vs PM) | 2 | 43.63 | 6.89 | 0.02 |
|  | Weight + Water Temp | 3 | 55.11 | 18.36 | 0 |
|  | Food Presence + Weight | 3 | 55.3 | 18.56 | 0 |
|  | Food Presence + Time of Day | 3 | 55.35 | 18.6 | 0 |
|  | Global | 8 | - | - |  |
| Medium energy | Water Temp | 2 | 277.7 | 0 | 0.32 |
|  | Weight + Water Temp | 3 | 278.1 | 0.46 | 0.25 |
|  | Air Temp | 2 | 280.2 | 2.54 | 0.09 |
|  | Weight | 2 | 280.8 | 3.13 | 0.07 |
|  | Null | 1 | 281.1 | 3.43 | 0.06 |
|  | Cohort Presence | 2 | 281.5 | 3.82 | 0.05 |
|  | Global | 8 | 282.3 | 4.62 | 0.03 |
|  | Food Presence + Weight | 3 | 283.2 | 5.51 | 0.02 |
|  | Food Presence | 2 | 283.3 | 5.66 | 0.02 |
|  | Time of Day (AM vs PM) | 2 | 283.4 | 5.71 | 0.02 |
|  | Food Presence + Time of Day | 3 | 285.7 | 8.04 | 0.01 |
| High energy | Weight + Water Temp | 3 | 305.4 | 0 | 0.41 |
|  | Time of Day (AM vs PM) | 2 | 307.4 | 2 | 0.15 |
|  | Air Temp | 2 | 308.1 | 2.76 | 0.1 |
|  | Water Temp | 2 | 308.2 | 2.78 | 0.1 |
|  | Weight | 2 | 308.9 | 3.51 | 0.07 |
|  | Food Presence + Time of Day | 3 | 309.7 | 4.37 | 0.05 |
|  | Null | 1 | 310.9 | 5.52 | 0.03 |
|  | Food Presence + Weight | 3 | 311.2 | 5.8 | 0.02 |
|  | Cohort Presence | 2 | 312.5 | 7.12 | 0.01 |
|  | Food Presence | 2 | 313.2 | 7.78 | 0.01 |
|  | Global | 8 | 313.7 | 8.36 | 0.01 |

Table S4. Candidate models for estimating covariate effects on energy expenditure of lesser scaup (*n* = 6) engaged in extremely low, low, medium, and high energy behaviors during respirometry trials at Patuxent Wildlife Research Center, Laurel, MD, October 2015–March 2016.

| Behavioral State | Model | k | AICc | Δ AIC | w |
| --- | --- | --- | --- | --- | --- |
| Extremely low energy (RMR) | Null | 1 | 67.06 | 0 | 0.61 |
|  | Time of Day (AM vs. PM) | 2 | 70.23 | 3.18 | 0.12 |
|  | Cohort Presence | 2 | 72.14 | 5.08 | 0.05 |
|  | Food Presence | 2 | 72.17 | 5.12 | 0.05 |
|  | Air Temp | 2 | 72.18 | 5.12 | 0.05 |
|  | Weight | 2 | 72.37 | 5.31 | 0.04 |
|  | Water Temp | 2 | 72.38 | 5.33 | 0.04 |
|  | Food Presence + Time of Day | 3 | 74.56 | 7.51 | 0.01 |
|  | Food Presence + Weight | 3 | 76.39 | 9.34 | 0.01 |
|  | Weight + Water Temp | 3 | 76.66 | 9.6 | 0 |
|  | Global | 8 | 135.25 | 68.19 | 0 |
| Low energy | Null | 1 | 232.02 | 0 | 0.19 |
|  | Water Temp | 2 | 232.13 | 0.11 | 0.18 |
|  | Air Temp | 2 | 232.59 | 0.57 | 0.14 |
|  | Time of Day (AM vs PM) | 2 | 232.68 | 0.66 | 0.13 |
|  | Cohort Presence | 2 | 234.02 | 2 | 0.07 |
|  | Food Presence | 2 | 234.04 | 2.02 | 0.07 |
|  | Weight | 2 | 234.27 | 2.25 | 0.06 |
|  | Weight + Water Temp | 3 | 234.55 | 2.53 | 0.05 |
|  | Food Presence + Time of Day | 3 | 234.89 | 2.87 | 0.04 |
|  | Food Presence + Weight | 3 | 236.46 | 4.43 | 0.02 |
|  | Global | 8 | 239.94 | 7.92 | 0 |
| Medium energy | Time of Day (AM vs PM) | 2 | 128.9 | 0 | 0.5 |
|  | Food Presence + Time of Day | 3 | 130.67 | 1.77 | 0.21 |
|  | Null | 1 | 132.19 | 3.29 | 0.1 |
|  | Air Temp | 2 | 133.64 | 4.74 | 0.05 |
|  | Food Presence | 2 | 133.75 | 4.85 | 0.04 |
|  | Water Temp | 2 | 134.93 | 6.03 | 0.02 |
|  | Weight | 2 | 134.96 | 6.06 | 0.02 |
|  | Cohort Presence | 2 | 134.98 | 6.08 | 0.02 |
|  | Food Presence + Weight | 3 | 136.84 | 7.94 | 0.01 |
|  | Weight + Water Temp | 3 | 138.07 | 9.17 | 0.01 |
|  | Global | 8 | 151.3 | 22.4 | 0 |
| High energy | Food Presence | 2 | 70.1 | 0 | 0.37 |
|  | Food Presence + Weight | 3 | 70.57 | 0.47 | 0.29 |
|  | Weight + Water Temp | 3 | 72.77 | 2.67 | 0.1 |
|  | Food + Time of Day | 3 | 73.73 | 3.63 | 0.06 |
|  | Water Temp | 2 | 73.86 | 3.76 | 0.06 |
|  | Null | 1 | 74.13 | 4.03 | 0.05 |
|  | Weight | 2 | 74.96 | 4.86 | 0.03 |
|  | Air Temp | 2 | 75.49 | 5.39 | 0.03 |
|  | Time of Day (AM vs PM) | 2 | 77.73 | 7.63 | 0.01 |
|  | Cohort Presence | 2 | 79.94 | 9.84 | 0 |
|  | Global | 8 | 290.47 | 220.38 | 0 |
